# Efficiency and Stability of Step-To Gait in Slow Walking

**DOI:** 10.3389/fnhum.2021.779920

**Published:** 2022-01-06

**Authors:** Kento Hirayama, Yohei Otaka, Taichi Kurayama, Toru Takahashi, Yutaka Tomita, Seigo Inoue, Kaoru Honaga, Kunitsugu Kondo, Rieko Osu

**Affiliations:** ^1^Faculty of Human Sciences, Waseda University, Saitama, Japan; ^2^Hatsutomi Hoken Hospital, Chiba, Japan; ^3^Department of Rehabilitation Medicine I, School of Medicine, Fujita Health University, Aichi, Japan; ^4^Department of Physical Therapy, Faculty of Health Sciences, Uekusa Gakuen University, Chiba, Japan; ^5^Faculty of Science and Technology, Keio University, Kanagawa, Japan; ^6^Tokyo Bay Rehabilitation Hospital, Chiba, Japan; ^7^Department of Rehabilitation Medicine, Juntendo University Graduate School of Medicine, Tokyo, Japan

**Keywords:** gait pattern, adaptation, efficiency, stability, rehabilitation

## Abstract

As humans, we constantly change our movement strategies to adapt to changes in physical functions and the external environment. We have to walk very slowly in situations with a high risk of falling, such as walking on slippery ice, carrying an overflowing cup of water, or muscle weakness owing to aging or motor deficit. However, previous studies have shown that a normal gait pattern at low speeds results in reduced efficiency and stability in comparison with those at a normal speed. Another possible strategy is to change the gait pattern from normal to step-to gait, in which the other foot is aligned with the first swing foot. However, the efficiency and stability of the step-to gait pattern at low speeds have not been investigated yet. Therefore, in this study, we compared the efficiency and stability of the normal and step-to gait patterns at intermediate, low, and very low speeds. Eleven healthy participants were asked to walk with a normal gait and step-to gait on a treadmill at five different speeds (i.e., 10, 20, 30, 40, and 60 m/min), ranging from very low to normal walking speed. The efficiency parameters (percent recovery and walk ratio) and stability parameters (center of mass lateral displacement) were analyzed from the motion capture data and then compared for the two gait patterns. The results suggested that step-to gait had a more efficient gait pattern at very low speeds of 10–30 m/min, with a larger percent recovery, and was more stable at 10–60 m/min in comparison with a normal gait. However, the efficiency of the normal gait was better than that of the step-to gait pattern at 60 m/min. Therefore, step-to gait is effective in improving gait efficiency and stability when faced with situations that force us to walk slowly or hinder quick walking because of muscle weakness owing to aging or motor deficit along with a high risk of falling.

## Introduction

**Figure 1:**
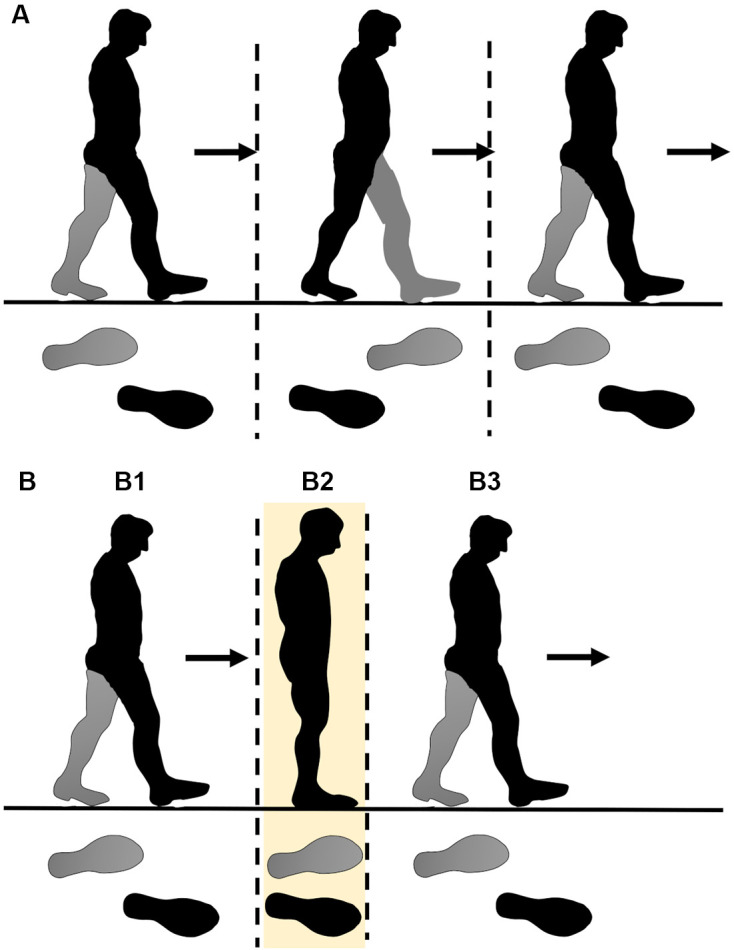
Schematic presentation of the gait patterns in the study. In normal gait **(A)**, the participant stepped with both legs symmetrically. In the step-to gait pattern **(B)**, the participant places the preceding leg, which is pre-determined to be either the left or right leg, by themselves at a certain place **(B1)**, and then swings the other leg to the location where the preceding leg is located **(B2)**. Subsequently, the participants swing the same preceding leg again **(B3)**.

People change their walking speed depending on situations (Leroux et al., 2002); they walk slowly when external environments are unstable, such as on slippery ice, or when internal environments are deficient in walking quickly, for example, weakened muscle owing to aging or motor deficit. In such cases, they can walk slowly with a normal gait pattern, or they can change their gait pattern to the step-to gait, in which the other foot is aligned with the first swing foot (Figure 1). For normal gait patterns, efficiency and stability have been reported to decrease at extremely low speeds (Cavagna et al., 1976; Bruijn et al., 2009; Murakami and Otaka, 2017; Best and Wu, 2020). However, no study has examined the characteristics of the step-to gait at significantly low speeds. If the step-to gait is stabler and more efficient than the normal gait at an extremely low speed, it can be recommended in situations where slow walking is necessary.

Cavagna et al. analyzed the percent recovery when healthy participants walked at various speeds with a normal gait pattern. The percent recovery is a measure of the gait efficiency, which is expressed as the rate of exchange of the potential and kinetic energies during walkingand can be estimated using the trajectory of the center of mass (CoM) measured by motion capture. They reported that the walking efficiency was maximum at intermediate walking speeds (66–116 m/min), which are the preferred walking speeds of healthy people, and showed an inverted U-shaped curve relative to the walking speed. In particular, walking efficiencies were found to be the highest at intermediate walking speeds and decreased as the walking speed decreased or increased (Cavagna et al., 1976).

The walk ratio, which is the ratio of the step length to cadence (step rate), is another important measure of human gait, and it is invariant across different speeds for a normal gait pattern (Sekiya, 1996). Zarrugh et al. (1974) reported that humans select a constant walk ratio across a wide range of speeds from all possible walk ratios because it is optimal in terms of energy consumption. However, Murakami et al. reported that this walk ratio was constant when the walking speed was moderate or fast but gradually increased when the speed was less than approximately 62 m/min. Therefore, they suggested that the efficiency is not optimized in a slow or normal gait (Murakami and Otaka, 2017).

Regarding the gait stability of normal gait, Best et al. analyzed the margin of stability of slow to very slow normal gait (6, 18, 30, and 36 m/min) using the lateral movement of the CoM and center of pressure of the trunk from open data measured on the treadmill. They reported that the margin of stability decreased with a decrease in speed, concluding that the lateral stability of normal gait decreased at low speeds, especially at extremely low speeds (Best and Wu, 2020).

In this study, we investigated the efficiency and stability of the step-to gait at five different speeds (i.e., 10 m/min, 20 m/min, 30 m/min, 40 m/min, and 60 m/min), ranging from very slow to the normal speed of a normal gait. We measured the percent recovery, walk ratio, and CoM lateral displacement in healthy participants during a treadmill walk.

The rest of the article is organized as follows. In sections “Materials and Methods”, we analyze the parameters (efficiency and stability) from motion capture data, and then compare the two gait patterns in detail. Section “Results” presents the results of the study. The percent recovery, walk ratio, and CoM lateral displacement in healthy participants during treadmill gait are presented. Finally, the discussion and conclusion are presented in section “Discussion”.

## Materials and Methods

### Participants

Eleven healthy adults participated in this study: five males {mean [standard deviation (SD)] age: 22.6 (0.5) years; mean weight: 61.8 (9.6) kg; and mean height: 170.0 (4.7) cm} and six females [mean (SD) age: 22.7 (0.5) years; mean weight: 50.3 (3.9) kg; and mean height: 161.5 (6.5) cm]. The number of participants was determined based on similar previous studies related to gait analysis of healthy participants (Brown and Mueller, 1998; Orendurff et al., 2004; Best and Wu, 2020). The participants did not have any neurological or orthopedic diseases. The study protocol was approved by the Ethics Committee of Tokyo Bay Rehabilitation Hospital (No. 53-3). All participants provided written informed consent before participating in the study.

### Tasks

The participants were instructed to walk on a treadmill (TRD-210, SAKAI Medical Co., Ltd., Tokyo, Japan) following two types of walking patterns, that is, step-to gait pattern and normal gait pattern, at five speed conditions (10, 20, 30, 40, and 60 m/min). All the participants first walked at all speed conditions with a normal gait pattern, and after taking a short break, they walked at all speed conditions with the step-to gait pattern. This setup was implemented to exclude the aftereffect of step-to gait on normal gait. The ordering of the speed condition was randomly determined for each participant to remove order bias. In each condition, participants were asked to walk 40 steps after their gait stabilized. In the step-to gait pattern condition, the participants were instructed to walk by placing the preceding leg at a certain place and swinging the other leg to the location where the preceding leg was located (Figure 1). Before the experiments, the participants selected the preceding leg that they preferred to swing first and adequately practiced the gait pattern. Thereafter, the preceding leg was not changed for any participant throughout the experiment.

**Figure 2:**
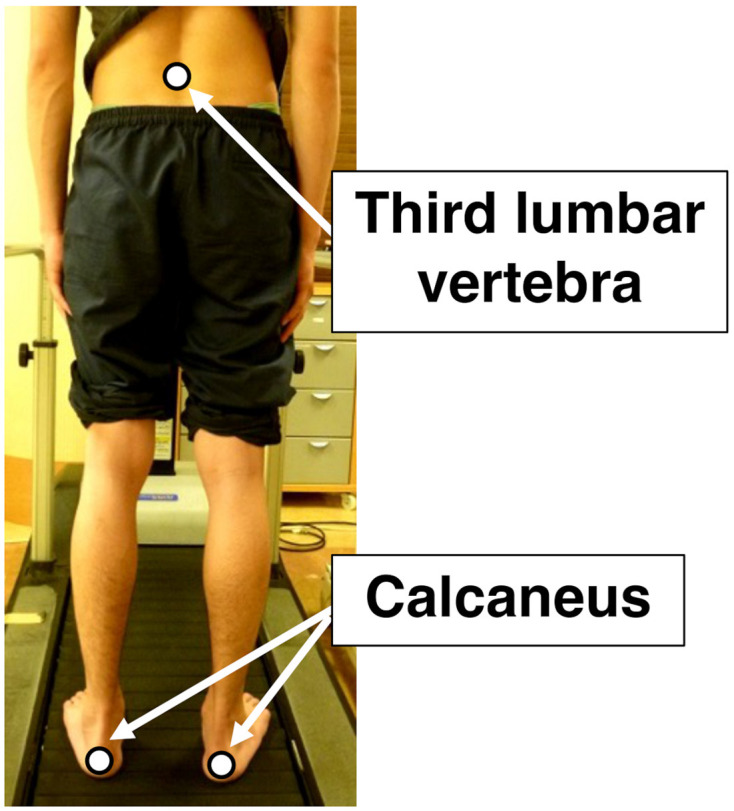
Positions of optical marker for the motion capture system. The white dots represent the positions of the optical markers. They are the third lumbar vertebra and the bilateral calcaneus. The movements of the markers were recorded from the backsides of the participants using the motion capture system.

### Motion Analysis

#### Measurements

The movements of both sides of the calcaneus and the spinous process of the third lumbar vertebra (Figure 2) were recorded at 100 Hz using a motion capture system (NDI OPTOTRAK, Waterloo, Ontario, Canada). After the motion data were low-pass filtered at 20 Hz using Butterworth fourth-order zero-lag filters, the efficiency and stability parameters were calculated using the data of 40 steps recorded after the gait stabilized during each task. Each step was determined using the vertical and lateral movements of both sides of the calcaneus. The movement of the third lumbar vertebra was recorded as the CoM movement (Meichtry et al., 2007; Kurayama et al., 2013). MATLAB R2013a (MathWorks Inc., Natick, MA, USA) was used for the motion analyses.

#### Parameters of Gait Efficiency

The percent recovery and walking ratio were calculated as the parameters of gait efficiency. Percent recovery is the rate of exchange of the potential and kinetic energies, focusing on the motion of the CoM during the walk (Cavagna et al., 1976). The larger the percent recovery, the less muscle activity is required during walking, and hence, the more efficient the gait. The percent recovery is approximately 65% at the intermediate walking speed of healthy people; thus, the remaining 35% depends mainly on the workload produced by muscle activity. We computed the percent recovery from the change in mechanical energy during each gait test using the CoM displacement data and the following formula (Cavagna et al., 1976).


%Recovery={1−WtWp+Wk}×100

Here, *Wt* is the increase in total mechanical energy, *Wp* is the increase in potential energy, and *Wk* is the increase in kinetic energy. The walk ratio was calculated by dividing the mean step length (m) by the cadence (steps/min). Each step was detected from each heel contact, which was the lowest point of motion data for each calcaneus.

#### Parameters of Gait Stability

The CoM lateral displacement was calculated as the parameter of gait stability. The CoM lateral displacement has been reported to be a useful parameter for evaluating the gait stability (Iida and Yamamuro, 1987; Shinoda et al., 2008). For the CoM lateral displacement, we calculated the difference between the maximum and minimum values of the lateral motion of the third lumbar vertebra in each gait cycle and then averaged them over 20 gait cycles. Furthermore, we calculated the CoM lateral displacement during the single-leg stance-phase: the preceding leg and following leg in the step-to gait, and the averaged values with left and right legs in the normal gait.

### Statistical Analysis

For all parameters, the effects of the pattern of GAIT (normal and step-to gait pattern), SPEED (five-speed conditions), and their interaction (GAIT×SPEED) were investigated using a two-way analysis of variance (ANOVA) with repeated measures. Furthermore, two-way ANOVAs were conducted for the CoM lateral displacement during the single-leg stance-phase in the step-to gait and that of the stance-phases with both left and right legs in the normal gait. When a statistically significant interaction was found, a paired t-test between gait patterns for each speed condition was performed as a *post hoc* analysis for GAIT; when a statistically significant main effect of SPEED was found, multiple comparisons with Shaffer correction were performed for SPEED between speed conditions for each gait pattern. Before each statistical analysis, the normality of the data was confirmed using the Shapiro-Wilk test. The spherical assumption was tested using Mauchly’s sphericity test, and the degrees of freedom were adjusted using the Greenhouse-Geisser method when the spherical assumption was rejected. In addition, we investigated the correlation between percent recovery and CoM vertical displacement using Spearman’s rank correlation coefficient to check the positive relationship between the CoM vertical displacement and percent recovery, similar to a previous study on normal gait (Wurdeman et al., 2017). Statistical analyses were performed using R (version 3.6.1.). The statistical significance was set at *P* < 0.05.

**Figure 3:**
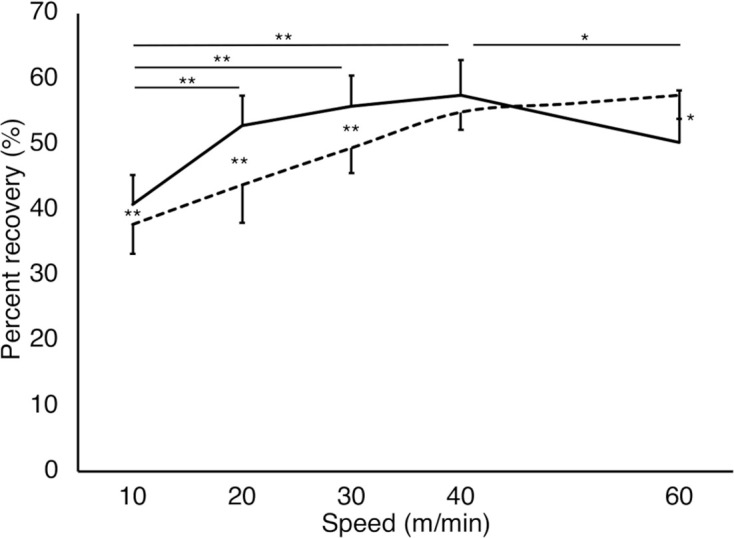
Percent recovery of each gait pattern at each speed condition. The solid and dashed lines represent the step-to gait and normal gait patterns, respectively. The vertical and horizontal axes represent the percent recovery (%) and gait speeds (m/min), respectively. A large percent recovery indicates a more efficient gait with less muscle activity. There were significant differences in normal gait for all speed conditions (*P* < 0.05; not shown in the figure). Significant differences were found in the step-to gait pattern between speed conditions as well as between the gait patterns, which are presented in the figure. The larger the value, the higher the mechanical energy exchange efficiency. **P < 0.01, *P < 0.05. Error bars: Standard deviation.

## Results

### Percent Recovery

A significant interaction (GAIT×SPEED) was observed in percent recovery (*F*_(1.78, 17.8)_ = 20.9, *P* < 0.01, partial *η*^2^ = 0.68; Figure 3). Significant main effects were observed in both GAIT (*F*_(1, 10)_ = 11.3, *P* < 0.01, partial *η*^2^ = 0.53) and SPEED (*F*_(1.91, 19.1)_ = 44.6, *P* < 0.01, partial *η*^2^ = 0.82). The tests of the simple main effect of the gait patterns revealed that the percent recovery was significantly larger for the step-to gait pattern than for the normal gait at 10 m/min, 20 m/min, and 30 m/min (*P* < 0.01), while it was significantly smaller for the step-to gait pattern than for the normal gait at 60 m/min (*P* = 0.012). The percent recovery was smaller at slower conditions than faster ones for both gait patterns, except at 60 m/min for the step-to gait pattern. The test of the simple main effect of the speeds showed simple main effects for normal (*P* < 0.01) and step-to gait (*P* < 0.01). For the normal gait pattern, multiple comparisons showed a significant difference between all speed conditions (*P* < 0.01). For the step-to gait pattern, multiple comparisons showed significant differences between 10 m/min and 20 m/min, 30 m/min and 40 m/min (*adj* *P* < 0.01), and 40 m/min and 60 m/min conditions (*adj* *P* = 0.019).

### Walk Ratio

**Figure 4:**
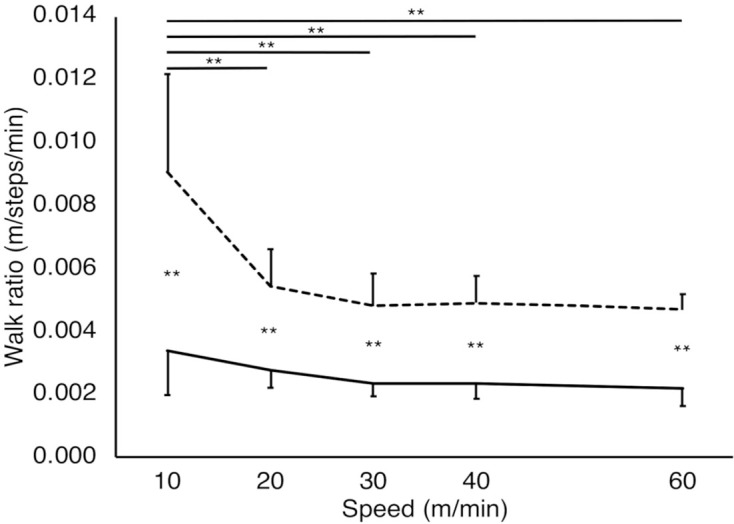
Walk ratio of each gait pattern at each speed condition. The solid and dashed lines represent step-to gait and normal gait patterns, respectively. The vertical and horizontal axes represent the walk ratio (m/steps/min) and gait speeds (m/min), respectively. The walk ratio in the normal gait pattern was significantly larger at 10 m/min than the other speed conditions (*P* < 0.01). The walk ratio for the step-to gait pattern was significantly smaller than that for normal gait at all speed conditions. **P < 0.01. Error bars: Standard deviation.

A significant interaction (GAIT×SPEED) was observed for the walk ratio (*F*_(1.6, 16.2)_ = 19.6, *P* < 0.01, partial *η*^2^ = 0.66; Figure 4). Significant main effects were observed in both GAIT (*F*_(1, 10)_ = 136.1, *P* < 0.01, partial *η*^2^ = 0.93) and SPEED (*F*_(1.19, 11.9)_ = 16.9, *P* < 0.01, partial *η*^2^ = 0.62). The simple main effect of the gait patterns revealed that the walk ratio for the step-to gait pattern was significantly smaller than that for the normal gait at all speed conditions (*P* < 0.01). A test of the simple main effect of the speeds showed simple main effects for the normal (*P* < 0.01) and step-to gait patterns (*P* = 0.026). Multiple comparisons between speed conditions of the normal gait pattern showed a significantly larger walk ratio at 10 m/min in comparison with the other speed conditions (*adj* *P* < 0.01). The walk ratio for the step-to gait pattern showed no statistically significant differences between all speed conditions.

**Figure 5:**
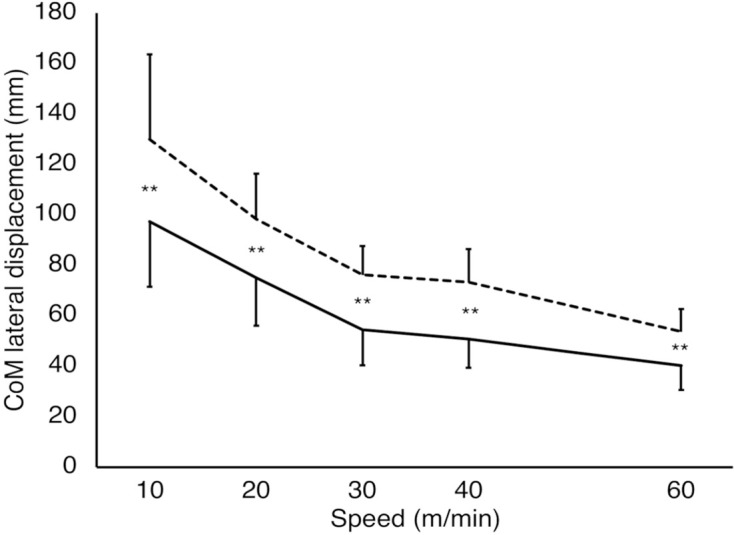
CoM lateral displacement of each gait pattern at each speed condition. The solid and dashed lines represent the step-to gait and normal gait patterns, respectively. The vertical and horizontal axes represent the CoM lateral displacement (mm) and gait speeds (m/min). The larger the CoM lateral displacement, the larger the gait instability. There were significant differences between all speed conditions, except those at 30 and 40 m/min in each gait pattern (*P* < 0.01; not shown in the figure). The CoM lateral displacement for the step-to gait pattern was significantly smaller than that for normal gait at all speed conditions (*P* < 0.01), which are shown in the figure. **P < 0.01. Error bars: Standard deviation.

### CoM Lateral Displacement

A significant interaction (GAIT×SPEED) was observed in the CoM lateral displacement (*F*_(4, 40)_ = 5.58, *P* < 0.01, partial *η*^2^ = 0.35; Figure 5). Significant main effects were observed in both GAIT (*F*_(1, 10)_ = 63.4, *P* < 0.01, partial *η*^2^ = 0.86) and SPEED (*F*_(4, 40)_ = 54.2, *P* < 0.01, partial *η*^2^ = 0.84). A test of the simple main effect of the gait patterns revealed that the CoM lateral displacements were significantly smaller for the step-to gait pattern than for the normal gait at all speed conditions (*P* < 0.01). A test of the simple main effect of the speeds showed simple main effects in both normal (*P* < 0.01) and step-to gait patterns (*P* < 0.01). Multiple comparisons between each pair of speed conditions showed that the CoM lateral displacements were significantly larger for the low-speed conditions than for fast ones, except for those between 30 m/min and 40 m/min conditions in both gait patterns (*adj* *P* < 0.01). To investigate why the CoM lateral displacement of the step-to gait was significantly smaller than that of the normal gait, we compared the CoM lateral displacement during the stance-phase of the following leg as well as the preceding leg in the step-to gait with that during the single-leg stance-phase in the normal gait. For the CoM lateral displacement of the following-leg during the stance-phase (Supplementary Figure 1A), the two-way ANOVA showed a significant interaction (GAIT×SPEED; *F*_(1.77, 17.72)_ = 10.89, *P* < 0.01, partial *η*^2^ = 0.52). The significant main effects were observed in both factors of GAIT (*F*_(1, 10)_ = 139.51, *P* < 0.01, partial *η*^2^ = 0.93) and SPEED (*F*_(1.83, 18.3)_ = 15.42, *P* < 0.01, partial *η*^2^ = 0.61). The significant simple main effects of GAIT were observed at 10 m/min (*P* < 0.05), 20 m/min, 30 m/min, 40 m/min, and 60 m/min (*P* < 0.01). Therefore, these results showed that the CoM lateral displacement of the following-leg during the stance-phase was significantly smaller than that during the single-leg stance-phase in the normal gait for all speed conditions. For the CoM lateral displacement of the preceding-leg during the stance-phase (Supplementary Figure 1B), the two-way ANOVA did not show a significant interaction (GAIT×SPEED; *P* = 0.06). The significant main effects were observed in both factors of GAIT (*F*_(1, 10)_ = 12.75, *P* < 0.01, partial *η*^2^ = 0.56) and SPEED (*F*_(1.4, 13.9)_ = 6.23, *P* < 0.05, partial *η*^2^ = 0.38).

### CoM Vertical Displacement

**Figure 6:**
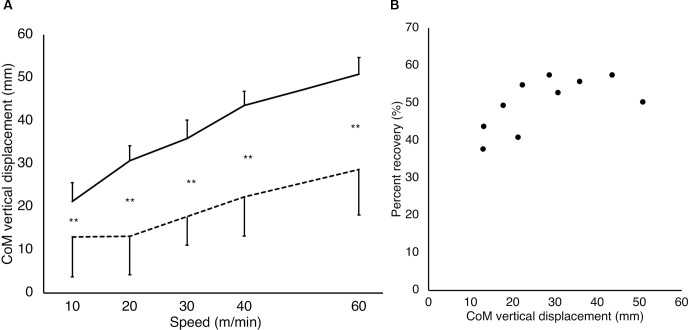
CoM vertical displacement and correlation between the CoM vertical displacement and percent recovery. For the CoM vertical displacement **(A)**, the solid and dashed lines represent the step-to gait and normal gait patterns, respectively. The vertical and horizontal axes represent the CoM vertical displacement (mm) and gait speeds (m/min), respectively. There were significant differences among all speed conditions, except those between 20 m/min and 30 m/min as well as 30 m/min and 40 m/min in the step-to gait pattern (*P* < 0.01; not shown in the figure). In the normal gait pattern, there were significant differences among all speed conditions, except those between 10 m/min and 20 m/min (*P* < 0.01; not shown in the figure). Significant differences were observed between the patterns, which are presented in the figure. **P < 0.01. Error bars: Standard deviation. For the correlation between the percent recovery and CoM vertical displacement **(B)**, the dots represent the averaged data of all participants in each condition with step-to gait and normal gait. The vertical and horizontal axes represent the percent recovery (%) and CoM vertical displacement (mm), respectively. A significant positive correlation was observed between the percent recovery and CoM vertical displacement using Spearman’s rank correlation coefficient (*ρ* = 0.721, *P* = 0.024).

A significant interaction (GAIT×SPEED) was observed in the CoMvertical displacement (*F*_(4, 40)_ = 7.50, *P* < 0.01, partial *η*^2^ = 0.43; Figure 6A). The significant main effects were observed in both GAIT (*F*_(1, 10)_ = 127.00, *P* < 0.01, partial *η*^2^ = 0.93) and SPEED (*F*_(4, 40)_ = 74.16, *P* < 0.01, partial *η*^2^ = 0.88). The tests of the simple main effect of the gait patterns revealed that the CoM vertical displacements were significantly larger for the step-to gait pattern than for the normal gait at all speed conditions (*P* < 0.01). The tests of the simple main effect of the speeds showed significant simple main effects in both normal (*P* < 0.01) and step-to gait patterns (*P* < 0.01). Multiple comparisons between each pair of speed conditions showed that the CoM vertical displacements were significantly smaller for the low-speed conditions than for thefast ones (*adj* *P* < 0.01), except those between 20 m/min and 30 m/min as well as 30 m/min and 40 m/min in the step-to gait pattern. In the normal gait pattern, the CoM vertical displacements were significantly smaller for the low-speed conditions than for fast ones (*adj* *P* < 0.01), except those between 10 m/min and 20 m/min. Furthermore, a significant positive correlation was observed between the percent recovery and CoM vertical displacement using Spearman’s rank correlation coefficient (*ρ* = 0.721, *P* = 0.024; Figure 6B).

## Discussion

This is the first study to investigate the efficiency and stability of the step-to gait relative to speeds and then compare them with those of normal gait. We used the percent recovery and walk ratio as parameters of gait efficiency, and CoM lateral displacement as a parameter of gait stability. The results suggest that the step-to gait is more stable at speeds between 10 m/min and 60 m/min and more efficient than normal gait at slow or very slow speeds of 10–30 m/min. Therefore, the results demonstrate the advantage of using step-to gait over normal gait when a slow or very slow walk is required, in terms of efficiency and stability.

Percent recovery is a measure of gait efficiency and is the exchange rate between the potential energy and kinetic energy (Cavagna et al., 1976). A large percent recovery indicates a more efficient gait with less muscle activity. In the speed range of 10–30 m/min, the percent recovery of the step-to gait was significantly larger than that of the normal gait, suggesting that step-to gait is an efficient gait pattern in slow or very slow walking. In healthy individuals with a normal gait, the percent recovery is maximal at intermediate walking speed (4 km/h to 7 km/h) and shows an inverse U-shaped relationship with speed variation (Cavagna et al., 1976). The percent recovery of the present results for the normal gait was maximal at 60 m/min, which is similar to the previous findings of Cavagna et al. (1976). Cappozzo et al. (1976) reported that the extension of the lower limbs during the stance phase of the gait cycle is important for accelerating the CoM using potential energy. As suggested by Wurdeman et al. (2017), we found a positive correlation between CoM vertical displacement and energy efficiency expressed as percent recovery (Figure 6B). In the present study, the CoM vertical displacement was significantly larger in the step-to gait than in the normal gait (Figure 6A). Thus, it is possible that in the step-to gait it was easier to extend the joints of the lower limbs and trunk and to raise the CoM than in the normal gait, as well as of the energy exchange rate of the CoM.

Many studies have shown that the walk ratio is unchanged among people walking at a wide range of walking speeds (Sekiya and Nagasaki, 1998). The walk ratio is optimal in terms of efficiency and attentional demands for a particular speed (Donelan et al., 2001; Legrand et al., 2021). However, in the present study, the walk ratio increased significantly at a very low speed of 10 m/min for the normal gait in comparison with other speeds of 20 m/min, 30 m/min, 40 m/min, and 60 m/min, while it remained constant in all speed conditions for the step-to gait. The observed increase in the walk ratio in slow normal gait was similar to the results of Murakami et al (Murakami and Otaka, 2017). Murakami et al. reported that in the normal gait of healthy participants, the walk ratio is consistent across the speed range of preferred to fast walking of 60–120 m/min; however, it increases with decreasing gait speed of 10–60 m/min. They argued that the coordination and automaticity of gait are disrupted during low-speed walking. Our results demonstrate that the step-to gait maintained a constant walk ratio even when the speed decreased, suggesting that the step-to gait is more coordinated and efficient than the normal gait at low speeds.

CoM lateral displacement is an important measure for evaluating gait balance and predicting falls (Tesio and Rota, 2019). The CoM lateral displacement of the step-to gait was significantly lower than that of the normal gait at all speeds, suggesting that the step-to gait is more advantageous in lateral stability than the normal gait. We speculate that the CoM lateral displacement was smaller in step-to gait than in normal gait because the CoM lateral displacement is smaller in step-to gait at the preceding leg’s stance phase following its stance phase with both legs aligned than at the stance phase in normal gait (Supplementary Figure 1). The CoM lateral displacement is the largest at the swing phase in normal gait (Perry and Burnfield, 1992). Furthermore, the lateral displacement of the CoM increased with decreasing speed for both gait patterns. The increase in CoM displacement with decreasing speed was similar to that in a previous study of normal gait (Orendurff et al., 2004).The results of the present study suggest that the CoM is easily maintained at the center of the body in the step-to gait pattern, and the risk of lateral fall for it is low compared with the normal gait.

This study has several limitations. In this experiment, the participants walked on the treadmill; thus, the findings of this study should be interpreted with caution because there are some differences between treadmill and ground walking (Alton et al., 1998). We estimated the efficiency using the CoM trajectory, but measurement of metabolic efficiency by exhaled gas is preferable for a more accurate evaluation of gait efficiency. Despite these limitations, the parameters of the present study can represent the efficiency and stability of gait; thus, the findings of this study are valuable in providing a new approach and hints for future research on gait with the step-to gait pattern.

The results of this study provide evidence that the step-to gait is advantageous, especially in terms of efficiency, for slow walking in an external environment with a high risk of falling (e.g., on slippery ice) or a physical function with a high risk of falling (e.g., aging). Furthermore, the results of this study prove the benefits of teaching step-to gait to patients who have gait disorders and walk at low speed during rehabilitation.In the future, we should investigate whether the characteristics of step-to gait in the elderly and patients with neurological and orthopedic diseases are similar to those of step-to gait in healthy participants in this study.

## Data Availability Statement

The raw data supporting the conclusions of this article will be provided by the authors, without undue reservation.

## Ethics Statement

The studies involving human participants were reviewed and approved by Tokyo Bay Rehabilitation Hospital. The participants provided their written informed consent to participate in this study. Written informed consent was obtained from the individual(s) for the publication of any potentially identifiable images or data included in this article.

## Author Contributions

KHi, YO, TK, and RO designed the experiments and prepared the manuscript. KHi, TK, and YT collected and analyzed the data. TT, SI, KHo, and KK provided important suggestions for data analysis and the manuscript. All authors contributed to the article and approved the submitted version.

## Conflict of Interest

The authors declare that the research was conducted in the absence of any commercial or financial relationships that could be construed as a potential conflict of interest.

## Publisher’s Note

All claims expressed in this article are solely those of the authors and do not necessarily represent those of their affiliated organizations, or those of the publisher, the editors and the reviewers. Any product that may be evaluated in this article, or claim that may be made by its manufacturer, is not guaranteed or endorsed by the publisher.
