# Supplementary material for: Efficiency and Stability of Step-To Gait in Slow Walking
Source: Front Hum Neurosci. 2022 Jan 6;15:779920. doi: 10.3389/fnhum.2021.779920 (PMC8770272; doi:10.3389/fnhum.2021.779920)
Supplement: Supplementary file 1 [file Image_1.pdf]

Supplementary Material

1 Supplementary Figure

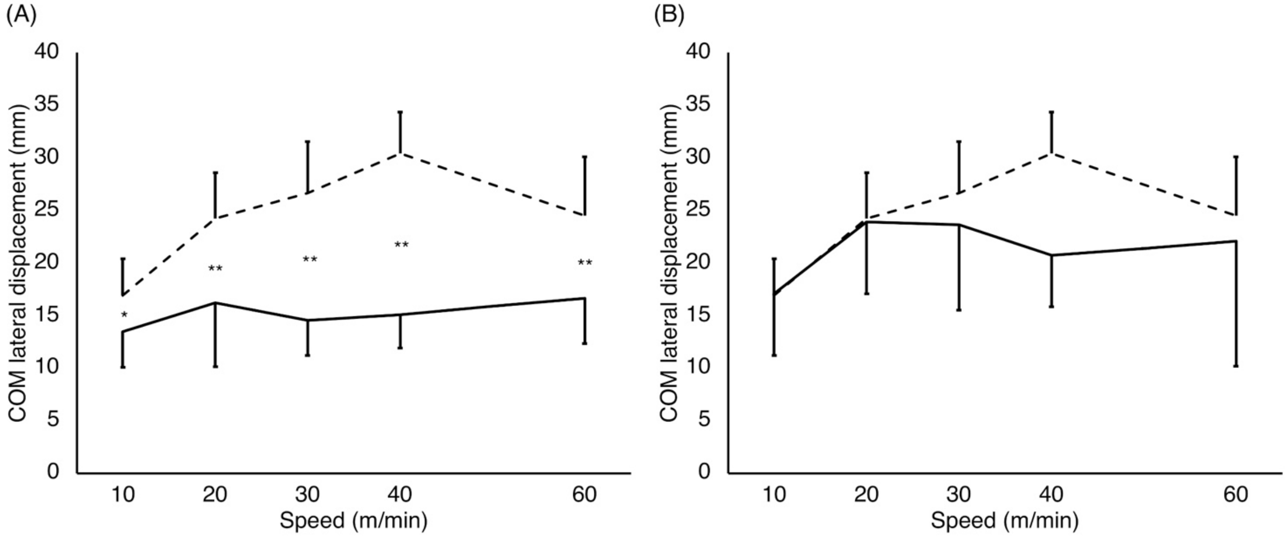

Supplementary Figure 1. CoM lateral displacement during single-leg stance-phase

The solid lines represent the CoM lateral displacement during the single-leg stance-phase of the following leg (A) and preceding leg (B) in the step-to gait. The dashed lines (A and B) represent the CoM lateral displacement during the single-leg stance-phase in the normal gait. The vertical and horizontal axes represent the CoM lateral displacement (mm) and gait speeds (m/min), respectively. For the CoM lateral displacement of the following-leg during the stance-phase (A), the two-way ANOVA showed a significant interaction (GAIT×SPEED) (F (1.77, 17.72) = 10.89, $P < 0.01$, partial $\eta^2 = 0.52$). The significant main effects were observed in both factors of GAIT (F (1, 10) = 139.51, $P < 0.01$, partial $\eta^2 = 0.93$) and SPEED (F (1.83, 18.3) = 15.42, $P < 0.01$, partial $\eta 2 = 0.61$). The significant simple main effects of GAIT were observed at 10 m/min ($P < 0.05$), 20 m/min, 30 m/min, 40 m/min, and 60 m/min ($P < 0.01$). Therefore, these results showed that the CoM lateral displacement of the following-leg during the stance-phase was significantly smaller than that during the stance-phase in the normal gait for all speed conditions. For the CoM lateral displacement of the preceding-leg during the stance-phase (B), the two-way ANOVA did not show a significant interaction (GAIT×SPEED) ($P = 0.06$). The significant main effects were observed in both factors of GAIT (F (1, 10) = 12.75, $P < 0.01$, partial $\eta^2 = 0.56$) and SPEED (F (1.4, 13.9) = 6.23, $P < 0.05$, partial $\eta 2 = 0.38$). **: $P < 0.01$ and *: $P < 0.05$. Error bars: Standard deviation.
